# MINDY1 Induces PD-L1 Deubiquitination to Promote Immune Escape in Hepatocellular Carcinoma by the Wnt/β-Catenin Pathway

**DOI:** 10.32604/or.2025.067638

**Published:** 2025-10-22

**Authors:** Xingchao Song, Qiuyu Song, Xiao Ma, Anzhi Xu, Chunyan Tian

**Affiliations:** 1 Department of Hepatobiliary and Pancreatic Surgery, The Affiliated Xuzhou Municipal Hospital of Xuzhou Medical University, Xuzhou, 221116, China; 2 Clinical Medical College, Tianjin Medical University, Tianjin, 300270, China

**Keywords:** Hepatocellular carcinoma, motif interacting with ubiquitin-containing novel DUB family-1, Wnt/β-catenin, programmed death-ligand 1, immune escape

## Abstract

**Background:**

Motif interacting with ubiquitin-containing novel DUB family-1 (MINDY1) could enhance the stability of programmed death-ligand 1 (PD-L1). The study aimed to investigate whether MINDY1 regulates the immune escape of hepatocellular carcinoma (HCC) mediated by PD-L1.

**Methods:**

MINDY1 and PD-L1 levels were detected through Western blot. The link between MINDY1 and PD-L1 was validated using the co-immunoprecipitation assay. The malignant biology of HCC cells was assessed through Cell Counting Kit-8, Carboxyfluorescein Succinimidyl Ester staining, transwell, and wound healing assay. CD8^+^ T cells were isolated and then co-cultured with HCC cells. Enzyme-linked immunosorbent Assay kits detected CD8^+^ T cytokine content. CD8^+^ T cell activation markers, PD-L1 ubiquitination levels, and Wnt/β-catenin pathway-associated protein levels were detected through Western blot. A HCC nude mouse model was developed, Ki-67 positivity and CD8^+^ T-cell infiltration were assessed through pathological staining and flow cytometry.

**Results:**

MINDY1 and PD-L1 levels were elevated in HCC. Overexpression of MINDY1 increased migrating and invading cells, elevated cell viability, and decreased apoptosis in HCC cells, leading to PD-L1 deubiquitination. Knockdown of MINDY1 reversed all of these indicators. Co-culturing with HCC cells overexpressing MINDY1 resulted in decreased proliferative capacity and cytotoxicity of CD8^+^ T cells, increased apoptosis, and decreased levels of cytokines and activation markers in CD8^+^ T cells. MINDY1 triggered Wnt/β-catenin pathway, Wnt activators further promoted PD-L1 deubiquitination and suppressed CD8^+^ T cell activation. MINDY1 overexpression increased PD-L1 and Ki67 positivity level in HCC tumors, suppressed CD8^+^ T-cell infiltration.

**Conclusion:**

MINDY1 promotes PD-L1 deubiquitination and inhibits CD8^+^ T cell activation by stimulating the Wnt/β-catenin pathway, consequently promoting HCC tumor immune escape.

**Highlights**

- MINDY1 expression is elevated in the tissues and cells of hepatocellular carcinoma (HCC).
- Overexpression of MINDY1 promotes HCC malignancy progression, while MINDY1 knockdown does the opposite.
- Overexpression MINDY1 upregulates PD-L1 expression, promotes PD-L1 deubiquitination, and inhibits CD8^+^ T cell activation.
- MINDY1 promotes PD-L1 deubiquitination and induces immune escape by activating the Wnt/β-catenin pathway.

## 1 Introduction

Primary liver cancer is the sixth most common type of cancer globally and the fourth most common reason for cancer-associated fatalities [1]. According to the latest American cancer statistics, 42,000 new cases are expected to occur in 2025, while 30,000 deaths will occur, with men having higher incidence and mortality rates than women [2]. In addition, China is a high-incidence country for liver cancer, among all types of cancer, liver cancer ranking fifth in incidence rates, and the mortality rate ranking second only to lung cancer [3]. The principal factors contributing to liver cancer are hepatitis B and alcoholic cirrhosis; in addition, aflatoxin exposure and liver fluke infection are also involved in liver cancer [4,5]. Hepatocellular carcinoma (HCC) represents the most predominant form of liver cancer, constituting 75%–85% of total cases, followed by cholangiocarcinoma [6,7]. HCC has an insidious disease onset, strong invasiveness, easy metastasis and recurrence, and its treatments include surgical resection, chemotherapy, immunotherapy, etc., yet the outcomes remain disappointing [8,9]. Currently, the mechanisms of HCC development and invasion, and metastasis are not fully understood. Thus, it is particularly important to explore invasive and metastatic mechanisms of HCC and to find effective therapeutic targets.

Ubiquitination, which includes protein degradation, protein-protein interactions, and DNA damage repair, is important for the post-translational modification process of proteins and is a vital part in the physiological activities of cells [10]. Ubiquitination is the mechanism in which a ubiquitin molecule covalently binds to particular lysine residues of a substrate protein in the presence of a series of ubiquitin-activating enzymes [11]. Like acetylation and phosphorylation, ubiquitination is a reversible reaction, the reverse of which is known as deubiquitination [12]. Abnormalities in ubiquitination are strongly linked with the advancement of numerous human disorders, like cancers and cardiovascular diseases [13,14]. Furthermore, ubiquitination is vital in regulating chemoresistance and radiosensitization in HCC [15,16]. Motif interacting with ubiquitin-containing novel DUB family-1 (MINDY1) is an important part of the deubiquitinating enzymes (DUBs) family, which is crucial for the maintenance of protein homeostasis, signaling, and disease development in cells [17]. MINDY1 has been shown to maintain the stability of programmed death-ligand 1 (PD-L1), thereby facilitating the immune escape of breast cancer [18]. Notably, in our previous study, MINDY1 has been found to contribute to PD-L1 deubiquitination, and MINDY1 is strongly linked with poor outcome in individuals with HCC [19]. However, the effect of MINDY1 on HCC immune escape and its specific mechanism are unknown.

Wnt/β-catenin classical pathway holds a pivotal position in cell biology, influencing a series of key processes like cell differentiation and proliferation [20,21]. A variety of investigations have demonstrated that this pathway is aberrantly activated in various malignant tumors, like HCC, colorectal cancer, and gastric cancer [22–24]. Wang et al. showed that suppression of Wnt/β-catenin pathway induced PD-L1 degradation, thereby facilitating immune cell infiltration and inhibiting cervical cancer progression [25]. In addition, once the Wnt/β-catenin pathway was activated, it caused a rise in the proliferation and migration of HCC cells, subsequently promoting the malignant progression of HCC [26]. Therefore, this study investigated whether MINDY1 affects the ubiquitination level of PD-L1 through regulating the aforementioned signaling pathway, thereby facilitating immune escape from HCC. This research aimed to elucidate the specific mechanism of action of MINDY1 in regulating the progression of HCC and to offer novel molecular targets for HCC therapy.

## 2 Materials and Methods

### 2.1 Bioinformatics Analysis

Query MINDY1 expression in primary HCC and normal tissues through The Cancer Genome Atlas (TCGA) database (https://www.cancer.gov/ccg/research/genome-sequencing/tcga (accessed on 01 July 2025)).

### 2.2 Clinical Tissue Sample

The samples of HCC tumor and adjacent tissues were collected from HCC patients (n = 45) who were treated at the Affiliated Xuzhou Municipal Hospital of Xuzhou Medical University. After being washed, tissues were placed in liquid nitrogen for future use. The Ethics Committee of the Affiliated Xuzhou Municipal Hospital of Xuzhou Medical University approved this research (No. xyyll [2025]119). Additionally, all patients involved signed the informed consent forms regarding sample collection.

### 2.3 Cell Culture and Processing

Hep3B, and Huh-7 cells, are widely used cell lines in HCC research, possessing excellent phenotypic and genotypic characteristics [27,28]. Therefore, we utilized these two cell lines for *in vitro* studies. Hep3B (CL-0102), Huh-7 (CL-0120), and human hepatocytes THLE-2 (CL-0833) were from Pricella Biotechnology (Wuhan, China), the Short Tandem Repeat (STR) identification was correct, and all cells are free of mycoplasma contamination. The above-mentioned cells were all cultured in Dulbecco’s Modified Eagle Medium (DMEM) complete medium (PM150210B, Pricella Biotechnology, Wuhan, China). The culture temperature was 37°C, with saturated humidity and 5% CO_2_ by volume. Once the cells achieved a confluence of 80% or more, subculture was carried out, and the subculture ratio was 1:3. The growth medium was refreshed every 3 days.

The MINDY1 overexpression plasmid (MINDY1), PD-L1 short hairpin RNA (sh-PD-L1), MINDY1 short hairpin RNA (sh-MINDY1), and their controls (Vector, sh-NC) were obtained from Sangon Biotech (Shanghai, China). In accordance with the guidelines for Lipofectamine 3000 (L3000001, Invitrogen, Carlsbad, CA, USA), the above-mentioned vectors were transfected into HCC cells, respectively. The cells were maintained in the incubator for a duration of 48 h, after which they were lysed with Radio Immunoprecipitation Assay (RIPA) lysis buffer (P0013B, Beyotime, Shanghai, China) to obtain proteins, and the transfection efficiency was assessed by measuring MINDY1 and PD-L1 protein levels.

Additionally, HCC cells (Hep3B and Huh-7 cells) transfected with MINDY1 were treated with the Wnt signaling pathway activator SKL2001 (20 nM, S793781, Macklin, Shanghai, China) for 24 h, and this group was denoted as the MINDY1+SKL2001 group [29]. HCC cells transfected with MINDY1 were exposed to the Wnt inhibitor LiCl (20 mM, HY-Y0649, MedChemExpress, Monmouth Junction, NJ, USA) and incubated for 24 h, and this group was denoted as the MINDY1+LiCl group [30].

### 2.4 Cell Counting Kit-8 (CCK-8) Assay

Hep3B and Huh-7 cell suspensions (100 μL) were seeded into 96-well cell culture plates, so that the number of cells in each well reached 1.0 × 10^4^ cells. The culture plates were placed in an incubator for the cells to adhere and grow. Subsequently, CCK-8 reagent (10%, C917226, Macklin, Shanghai, China) was thoroughly mixed with the cells and then incubated for 2 h. The OD_450_ value was detected through a 1410101 microplate reader (Thermo Fisher Scientific, Waltham, MA, USA) to evaluate cell viability.

### 2.5 Transwell Assay

Trypsin (0.25%, HY-129047) and Matrigel (HY-K6002) were obtained from MedChemExpress (Monmouth Junction, NJ, USA). Hep3B and Huh-7 cells were digested with trypsin to prepare single-cell suspensions. For invasion assay, Matrigel was diluted with medium (without serum), and the diluted gel (100 μL) was drawn up and plated onto the bottom of each Transwell (8 μm, Corning, Tewksbury, MA, USA). The next day, cell suspension (1.0 × 10^5^ cells, 100 μL) was inoculated into the small chamber, and the lower chamber was filled with 800 μL of culture medium, followed by culturing for 48 h. After discarding the culture solution, washed 3 times with PBS (0.1%, pH = 7.2), and the non-invasive cells were gently scraped off. Then chambers were placed in 4% paraformaldehyde (P885233, Macklin, Shanghai, China) for a 30-min fixation step, followed by staining with crystal violet (0.1%, C916088, Macklin, Shanghai, China) for 15 min. Subsequently, it was rinsed twice with PBS and left to air-dry naturally for 2 h. Randomly selected microscopic fields in each well were imaged using a DM3000 microscope (Leica, Heidelberg, Germany).

In the Transwell migration test, there was no need to add Matrigel to the chamber, and the remaining operation and analysis steps were mirrored those of the invasion assay.

### 2.6 Wound Healing Assay

An appropriate amount of Hep3B and Huh-7 cell suspensions (1.0 × 10^5^ cells) was introduced into a 6-well plate and cultured until the cells were completely adherent and formed a single layer. Using a 20-μL sterile pipette tip to make a perpendicular line across the edge of each well on the cell monolayer, and keep the width of the scratch uniform. Rinsed twice with PBS (0.1%, pH = 7.2) to remove the cell debris generated during the scratching process, and then placed back in the incubator for culturing. The scratch healing was monitored under a microscope at 0 h and 48 h post-scratching, and the width of the scratch area was statistically analyzed by ImageJ 1.54 h software (Wayne Rasband, National Institute of Mental Health, Bethesda, MD, USA).

### 2.7 Isolation of Peripheral Blood CD8^***+***^ T Cells

Venous blood samples (20 mL) were obtained from healthy blood donors (from Affiliated Xuzhou Municipal Hospital of Xuzhou Medical University). After dilution with PBS (0.1%, pH = 7.2), Ficoll separation medium (F4375, Sigma-Aldrich, St. Louis, MO, USA) was added. Following low-speed centrifugation, the buffy layer at the boundary of the plasma and the separation medium was aspirated. This layer of buffy coat was the peripheral blood mononuclear cells (PBMCs), washed the cells with PBS to remove impurities, and then a CD8^+^ T cell sorting kit (11348D, Invitrogen, Carlsbad, CA, USA) was utilized to separate CD8^+^ T cells. PBMCs were mixed with PBS, supplemented with biotin-labeled antibody (10 μL), and left to incubate for 10 min at 4°C. Then, PBS (30 μL) and CD8^+^ T cell microsphere antibody (20 μL) were added. After mixing, the sample was incubated at 4°C for 10 min. Following the guidelines provided in the kit, cells adsorbed on the magnetic separation column were isolated, which were the enriched CD8^+^ T cells. HCC cells transfected with various plasmids were cultured with a 3-fold number of CD8^+^ T cells in Transwell chambers for 48 h [31]. Additionally, the CD8^+^ T cells after co-culture were collected and mixed well with a Phycoerythrin (PE)-labeled tumor necrosis factor-α (TNF-α) antibody (12-7349-41, 1:25, Invitrogen) and a Fluorescein Isothiocyanate (FITC)-labeled perforin antibody (11-9994-42, 1:100, Invitrogen), and then incubated for half an hour [32]. The fluorescence intensity was evaluated through a flow cytometer (BD FACSCalibur^TM^, BD Biosciences, San Jose, CA, USA), and with CD8^+^ T cell proportion was evaluated by FlowJo v10.8 software (BD Biosciences).

### 2.8 Carboxyfluorescein Succinimidyl Ester (CFSE) Staining

The CFSE probe (5 μM, C1031, Beyotime, Shanghai, China) was introduced into the Hep3B, Huh-7, or CD8^+^ T cell suspension, then incubated at 37°C for labeling for 10 min [33]. The staining reaction was terminated by adding a five-fold volume of medium. The cells were then collected by centrifugation and washed two times with PBS (0.1%, pH = 7.2) to clear away the unbound CFSE. The success of the labeling was verified by observing a small aliquot of the labeled cell suspension under a M5000 fluorescence microscope (Invitrogen, Carlsbad, CA, USA). After 48 h of culture, CFSE intensity was measured using a flow cytometer. After culturing for 48 h, the CFSE intensity was assessed through a flow cytometer.

### 2.9 Cell Apoptosis Assay

Annexin V-FITC/propidium iodide (PI) apoptosis detection kit (HY-K1073, MedChemExpress, Monmouth Junction, NJ, USA) was employed to determine the cell apoptosis rate. After different treatments, Hep3B, Huh-7 cells or CD8^+^ T cells (1.0 × 10^6^ cells) were washed twice using PBS (0.1%, pH = 7.2), and mixed well with Binding Buffer (500 μL). Subsequently, 5 μL of Annexin-V-FITC and 5 μL of PI were added. Following thorough mixing, incubated in darkness for 15 min, and the apoptosis rate was then measured via a flow cytometer.

### 2.10 Lactate Dehydrogenase (LDH) Assay

The LDH assay Kit (C0016, Beyotime, Shanghai, China) was employed to assess cell toxicity [34]. CD8^+^ T cells were incubated alongside Hep3B or Huh-7 cells for 48 h. Subsequently, the mixture underwent centrifugation, and the cell supernatant was carefully aspirated. Next, 150 μL of LDH release reagent was added and thoroughly blended with the cells, incubated for another hour. 120 μL of supernatant was taken and mixed thoroughly with the LDH detection working solution (60 μL). After incubating for 30 min, the OD_490_ value of the samples was examined through a microplate reader to calculate the cytotoxicity.

### 2.11 Enzyme-Linked Immunosorbent Assay (ELISA)

CD8^+^ T cell activation was evaluated using human interferon-gamma (IFN-γ) (PI511, Beyotime, Shanghai, China) and human interleukin-2 (IL-2) ELISA Kit (PI580, Beyotime, Shanghai, China) [32]. Added the supernatant of the co-culture medium of CD8^+^ T cells and HCC cells (Hep3B and Huh-7) into the ELISA plate and incubated for 2 h, and then exposed to the corresponding antibody (100 μL) for 1 h. Next, horseradish peroxidase-labeled Streptavidin (100 μL) and the solution was gently shaken to mix evenly and incubated for 20 min. Following three washes with PBS (0.1%, pH = 7.2), the chromogenic agent 3,3^′^,5,5^′^-Tetramethylbenzidine (TMB) solution (0.1%) was added according to the instructions and kept in the dark at 37°C for 10 min. The termination solution was then introduced and mixed thoroughly, followed by measuring the OD_450_ value.

### 2.12 Subcutaneous Tumor in Nude Mice

Male Balb/c nude mice (4–5 weeks old, 10–15 g) were obtained from Vitalriver (Beijing, China) and placed in a clean-grade animal house for a one-week acclimation period. Housing conditions included a constant temperature of 22°C, relative humidity from 45% to 55%, and alternating light and dark for 12 h per day. The facilities were disinfected regularly. The nude mice were assigned randomly to seven distinct groups (n = 5). A 200-μL suspension of Hep3B cells (5.0 × 10^6^ cells/mice) transfected with MINDY1, Vector, sh-PD-L1, sh-NC, Vector+sh-NC, MINDY1+sh-NC, or MINDY1+sh-PD-L1 was administered subcutaneously into the right axillary region of mice, respectively. The size of the tumors was examined using a vernier caliper every week. During the test period, the animals’ appetite, activity status and abnormal behaviors were observed and recorded daily. The mice were euthanized through cervical dislocation on the 28th day. The mice were recognized as dead when they were observed to have no voluntary respiration, cardiac arrest, loss of corneal reflexes, flaccid limb muscles and no response to painful stimuli. There were no animals that died halfway or were euthanized early due to health problems. Tumor tissues were completely dissected using tissue scissors, and the tumor weights were recorded and photographed. Approval for the animal research was obtained from the Experimental Animal Ethics Committee of the Affiliated Xuzhou Municipal Hospital of Xuzhou Medical University (No. xyyll [2025]119(A01)).

### 2.13 Immunohistochemistry

Mouse tumor tissues were exposed to 4% paraformaldehyde for fixation. After that, they were embedded in paraffin and prepared into ultra-thin sections (4–5 μm). These sections were then placed in xylene (247642, Sigma-Aldrich, St. Louis, MO, USA) for dewaxing. These sections were hydrated in gradient ethanol, immersed in antigen retrieval solution (0.1 mol/L citric acid), and the antigens were retrieved by microwave. Then, tissues were immersed in 3% H_2_O_2_ solution, rinsed using PBS (0.1%, pH = 7.2), and then evenly covered with 5% bovine serum albumin (BSA, 37520, Thermo Fisher Scientific, Waltham, MA, USA) for blocking. Ki67 primary antibody (MA5-14520, 1:100, Invitrogen, Carlsbad, CA, USA) was added dropwise to the surface of tissue sections, and then kept at 37°C for 1.5 h. After that, covered with horseradish peroxidase-labeled secondary antibody (31430, 1:500, Invitrogen, Carlsbad, CA, USA) and incubated for 30 min. 3,3^′^-Diaminobenzidine (DAB; 0.05%, P0203, Beyotime, Shanghai, China) was employed for color development, and distilled water was used to terminate. Hematoxylin (0.5%, 51275, Sigma-Aldrich, St. Louis, MO, USA) was re-stained, rinsed with distilled water, and sealed using neutral balsam (C0173, Beyotime, Shanghai, China), then visualized using a fluorescence microscope.

### 2.14 Immunofluorescence

For the tumor tissue paraffin sections, they were deparaffinized and hydrated routinely, and then antigen retrieval was performed. 0.3% Tritonx-100 (T824275, Macklin, Shanghai, China) was dropped on the surface of the tissue sections for permeabilization for 10 min. After a 30-min incubation with 5% BSA to block, treated at 4°C with CD8 primary antibody (MA1-145, 1:50) for 12 h. On the subsequent day, incubated at 37°C with FITC-labeled secondary antibody (65-6111, 1:2000) for 1 h. The above antibodies were obtained from Invitrogen (Carlsbad, CA, USA). Subsequently, 4^′^,6-Diamidino-2-Phenylindole (DAPI) staining solution (C1005, Beyotime, Shanghai, China) was added, and then incubated in the dark for 10 min; the development was observed through a fluorescence microscope.

### 2.15 CD8^***+***^ T Cell Marker Detection

The tumor tissues of mice were taken and thoroughly ground to prepare a single-cell tumor suspension (1.0 × 10^7^/mL). Added a PE-labeled TNF-α antibody (12-7349-41, 1:25) and a FITC-labeled IFN-γ antibody (53-7319-42, 1:100). The above antibodies were obtained from Invitrogen (Carlsbad, CA, USA). After incubating for 30 min in darkness, the mixture was transferred to a flow cytometer, and the CD8^+^ T cell proportion was analyzed and counted by FlowJo software.

### 2.16 Ubiquitination Assay

Referring to Fu et al. [35], PD-L1 ubiquitination level was detected. Hep3B and Huh-7 cells transfected with Vector or MINDY1 were exposed to cycloheximide (CHX, 100 μg/mL, 398241, Sigma-Aldrich, St. Louis, MO, USA) for 0, 4, 8, and 12 h, respectively, or treated with the proteasome inhibitor MG-132 (40 ng/μL, HY-13259, MedChemExpress, Monmouth Junction, NJ, USA) for 6 h. HCC cells were treated with RIPA lysis buffer for 1 h. The resulting lysate supernatant was mixed with anti-PD-L1 (PA5-28115, 1:500) at 4°C for 2 h, with immunoglobulin G (IgG, MA5-42729, 1:100) as a control. The above antibodies were obtained from Invitrogen (Carlsbad, CA, USA). Subsequently, incubated with Anti-Hemagglutinin (HA) immunomagnetic beads (B26201, Selleck, Shanghai, China) at 4°C for 12 h. After centrifugation, the immunoprecipitated complexes were harvested, rinsed with buffer, resuspended in SDS-PAGE loading buffer (P0015, Beyotime, Shanghai, China), subjected to heating at 95°C for 5 min, and subsequently evaluated via Western blot to detect PD-L1 ubiquitination.

### 2.17 Co-Immunoprecipitation (Co-IP) Assay

Using a Classic Magnetic IP/Co-IP Kit (88804, Thermo Fisher Scientific, Waltham, MA, USA) to verify the interaction between MINDY1 and PD-L1 [32]. The transfection of HA-MINDY1 and Flag-PD-L1 was performed on Hep3B and Huh-7 cells for 24 h, then the cells were treated with cell lysis buffer. The cell lysate supernatant (200 μL) was mixed well with the specific antibody at 4°C for 12 h. Then, added protein A/G agarose beads and incubated for 2 h to allow the antibody-antigen complex to bind to the agarose beads. Following centrifugation (500× *g*, 5427R, Eppendorf, Hamburg, Germany), it was washed twice with buffer, mixed with SDS-PAGE loading buffer, heated at 95°C for a duration of 10 min, and then processed for Western blot.

### 2.18 Western Blot

Cells (Hep3B, Huh-7, and THLE-2), human tissues, or animal tissues following various treatments were lysed thoroughly using RIPA lysis buffer on ice to extract proteins. Then, proteins were separated via SDS-PAGE gels (10%), then transferred to a Polyvinylidene Fluoride (PVDF) membrane (Invitrogen, Carlsbad, CA, USA), and blocked with 5% BSA for 3 h. Following washing the membrane, it was exposed to primary antibodies at 4°C overnight, including anti-MINDY1 (PA5-113220, 1:1000, Invitrogen, Carlsbad, CA, USA), anti-PD-L1 (PA5-28115, 1:500, Invitrogen), anti-β-catenin (71-2700, 1:100, Invitrogen), anti-p-GSK3β (44-604G, 1:1000, Invitrogen), anti-GSK3β (710132, 1:200, Invitrogen), anti-Perforin (PA1-22489, 1:1000, Invitrogen), or anti-IL-2 (701080, 1:200, Invitrogen). The subsequent day, the membranes were treated with secondary antibody (31460, 1:20000, Invitrogen) for 2 h. The ECL luminescent solution (P0018S, Beyotime, Shanghai, China) was evenly dropped on the membrane and scanned using the iBright CL1500 gel imaging system (Invitrogen, Carlsbad, CA, USA). Using GAPDH (MA5-35235, 1:50000, Invitrogen) as an internal reference, after processing the images through ImageJ software, the grayscale values for each protein band were determined, and the relative expression of the protein was calculated.

### 2.19 Statistical Analysis

Every experiment was performed at least three times, and the findings were presented as mean ± standard deviation. Statistical analyses were performed using IBM SPSS Statistics version 26.0 software (Armonk, NY, USA). The various groups were assessed through one-way ANOVA to perform multiple comparisons. In cases of independent samples, if they were normally distributed, Student’s *t*-test was applied; otherwise, non-parametric tests were used. Plots were drawn using Prism software (Graphpad 9.0, San Diego, CA, USA). *p* < 0.05 represents a significant difference.

## 3 Results

### 3.1 MINDY1 Is Elevated in HCC

**Figure 1:**
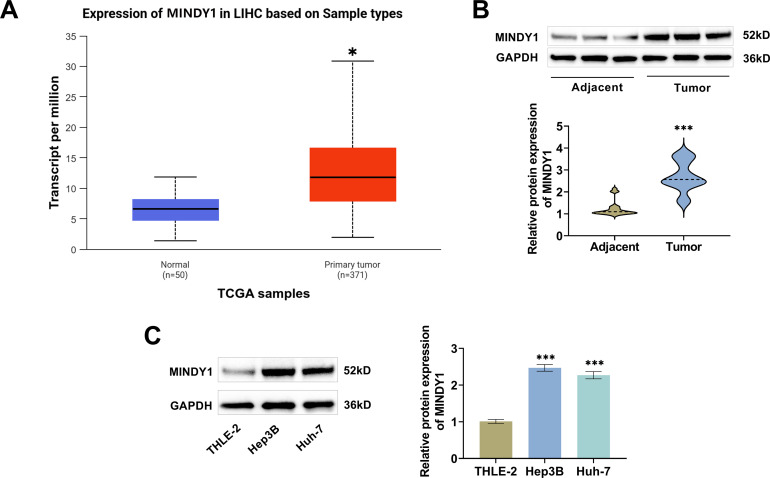
MINDY1 is elevated in HCC. (**A**) The TCGA database exhibited that MINDY1 level was higher in HCC tissues. (**B**) Western blot examined a high level of MINDY1 protein in the tumor samples of HCC patients (n = 45). (**C**) Western blot examined low level of MINDY1 in THLE-2 cells and high level in HCC cells (n = 3). *p < 0.05, ***p < 0.001

MINDY1 expression in 371 primary HCC tissues and 50 normal tissues was obtained through the TCGA database, with the MINDY1 level in HCC markedly elevated than normal tissues (Fig. 1A). Next, we detected MINDY1 levels in tissue samples from HCC patients by Western blot. Tumor samples exhibited a marked increase in MINDY1 expression, which corroborated the data shown in the TCGA database (Fig. 1B). Additionally, we also detected MINDY1 level in normal hepatocytes (THLE-2) and HCC cells (Hep3B and Huh-7). Consistent with the above results, MINDY1 was significantly elevated in HCC cells (Fig. 1C). These findings suggested that MINDY1 might be implicated in the malignant progression of HCC.

### 3.2 Overexpression of MINDY1 Promotes HCC Malignancy Progression

**Figure 2:**
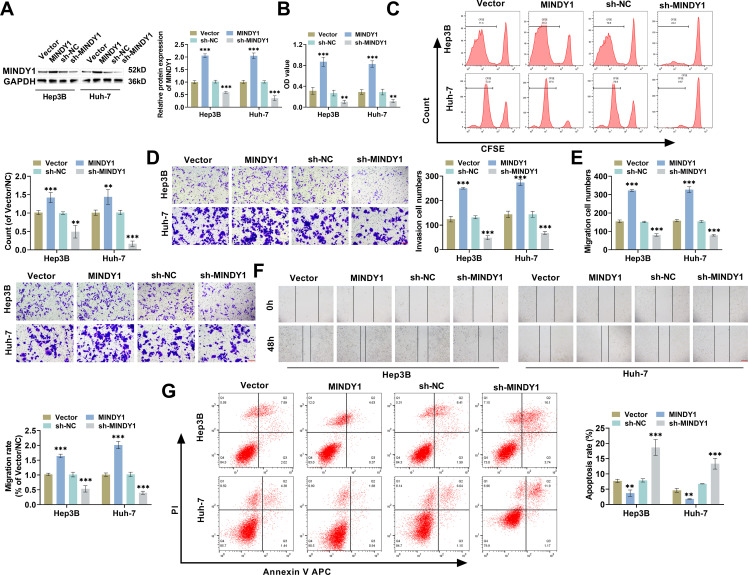
Overexpression of MINDY1 promotes HCC malignancy progression. (**A**) MINDY1/Vector and sh-MINDY1/sh-NC were transfected, and western blot demonstrated the successful overexpression/knockdown of MINDY1 in HCC cells. (**B**) CCK-8 assay confirmed that Hep3B and Huh-7 cell viability was increased after MINDY1 transfection, while it was decreased after sh-MINDY1 transfection. (**C**) CFSE staining confirmed that MINDY1 transfection led to enhanced proliferation ability of HCC cells, and sh-MINDY1 transfection led to reduced proliferation ability. (**D**,**E**) Transwell assays confirmed that invasive and migratory cell numbers were increased after MINDY1 transfection, and decreased after sh-MINDY1 transfection (20×, 100 μM). (**F**) Wound healing assay examined the migration ability of HCC cells at 0 and 48 h (10×, 200 μM). (**G**) Flow cytometry revealed HCC cell apoptosis rate was decreased after MINDY1 transfection, and increased after sh-MINDY1 transfection. n = 3. **p < 0.01, ***p < 0.001

To explore the influence of MINDY1 level on HCC progression, Vector/MINDY1 and sh-NC/sh-MINDY1 were transfected into HCC cells. After MINDY1 transfection, MINDY1 level was increased remarkably, while sh-MINDY1 transfection led to a significant decrease in MINDY1 expression. This indicated that the transfection of MINDY1 or sh-MINDY1 can effectively regulate MINDY1 level, allowing for subsequent functional assays (Fig. 2A). CCK-8 assay indicated that MINDY1 overexpression markedly elevated HCC cell viability, whereas MINDY1 silencing caused a marked reduction in cell viability (Fig. 2B). CFSE, a fluorescent dye capable of penetrating the cell membrane, is used to analyze cell proliferation. CFSE staining results showed that CFSE-positive cell numbers were increased significantly after MINDY1 overexpression, while MINDY1 knockdown reduced cell proliferation ability (Fig. 2C). Transwell assay findings indicated that invasive and migratory HCC cells was increased significantly after MINDY1 overexpression, but MINDY1 knockdown hindered cell invasion and migration (Fig. 2D,E). Wound healing assay also demonstrated that the wound healing rate was elevated markedly after MINDY1 overexpression, whereas MINDY1 knockdown suppressed cell migration (Fig. 2F). Moreover, after MINDY1 transfection, cell apoptosis rates were decreased significantly, while sh-MINDY1 transfection caused a marked rise in the apoptosis rate (Fig. 2G). Overall, these results suggested that MINDY1 overexpression promoted HCC malignancy progression, while MINDY1 knockdown inhibited malignancy progression.

### 3.3 Overexpression of MINDY1 Promotes PD-L1 Deubiquitination and Upregulates PD-L1 Expression

**Figure 3:**
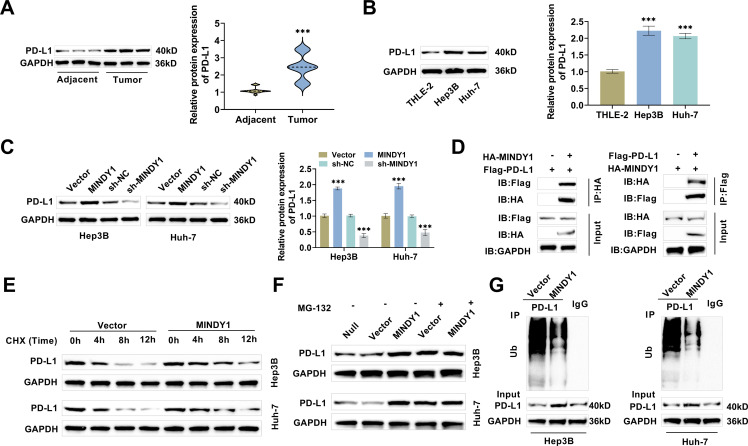
Overexpression of MINDY1 boosts PD-L1 level and promotes PD-L1 deubiquitination. (**A**) Western blot assessed that PD-L1 protein was heightened in tumor samples of HCC patients (n = 45). (**B**) Western blot detected that PD-L1 was poorly expressed in THLE-2 cells and highly expressed in HCC cells. (**C**) Western blot measured that transfection of MINDY1 increased PD-L1 protein expression, whereas transfection of sh-MINDY1 decreased PD-L1 levels. (**D**) The Co-IP results showed that MINDY1 could be co-precipitated with PD-L1. (**E**) PD-L1 protein level was detected through Western blot after 4, 8, and 12 h of CHX treatment. (**F**) PD-L1 level in HCC cells was examined through Western blot after 6 h of treatment with proteasome inhibitor (MG-132). (**G**) Western blot detection of PD-L1 ubiquitination level after MINDY1 overexpression. n = 3. ***p < 0.001

PD-L1 is an immune cell infiltration and immune checkpoint that inhibits T cell activation and leads to the formation of a suppressive immune microenvironment [36,37]. Therefore, we assessed PD-L1 levels in paracancerous normal tissues (Adjacent) and HCC tissues, and with PD-L1 level was notably increased in cancer tissues (Fig. 3A). It was also greater in Hep3B and Huh-7 cells than in THLE-2 cells (Fig. 3B), implying that high expression of PD-L1 might be implicated in HCC progression. Next, we transfected Vector/MINDY1 and sh-NC/sh-MINDY1 in HCC cells to explore the impact of deubiquitinating enzyme MINDY1 on PD-L1 expression. MINDY1 transfection led to a marked rise in PD-L1 level, while sh-MINDY1 transfection resulted in a marked decline, indicating that these transfections effectively regulate PD-L1 expression (Fig. 3C). Co-IP findings indicated a direct interaction between MINDY1 and PD-L1, suggesting that MINDY1 may regulate PD-L1 stability through post-translational modification (Fig. 3D). Overexpression of MINDY1 notably increased the half-life of PD-L1 in HCC cells after CHX treatment (Fig. 3E). In contrast, treatment with the proteasome inhibitor MG-132 eliminated the upregulation of MINDY1 on PD-L1, suggesting that MINDY1 may maintain the stability of PD-L1 by hindering the proteasome degradation pathway (Fig. 3F). Additionally, ubiquitination assays revealed that overexpression of MINDY1 notably diminished the ubiquitination level of PD-L1, suggesting that MINDY1 may indirectly reduce ubiquitination accumulation by suppressing the degradation of PD-L1 (Fig. 3G). These results demonstrated that overexpression of MINDY1 enhanced PD-L1 stability through promoting PD-L1 deubiquitination, thereby upregulating the PD-L1 level.

### 3.4 Overexpression of MINDY1 Hinders CD8^***+***^ T Cell Activation

**Figure 4:**
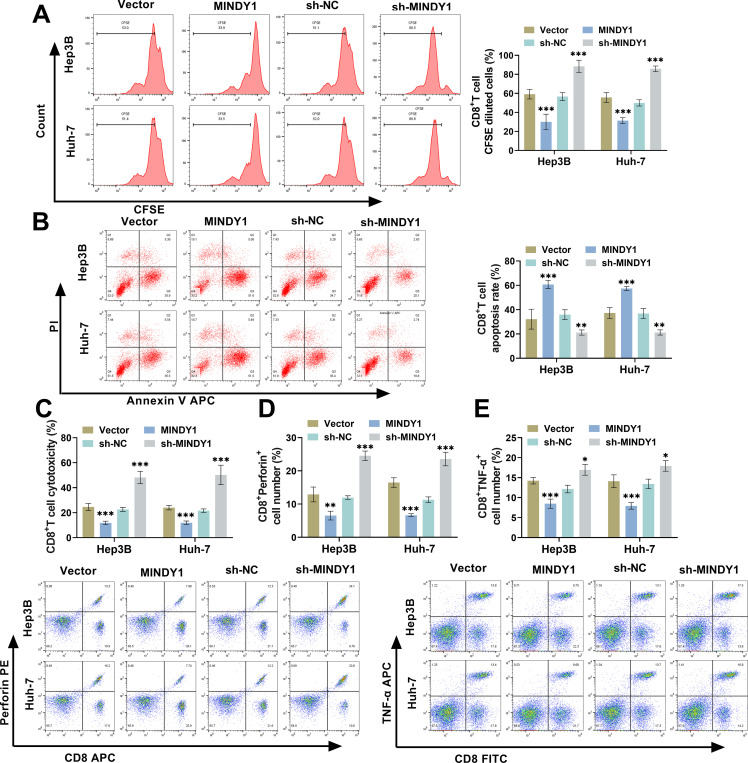

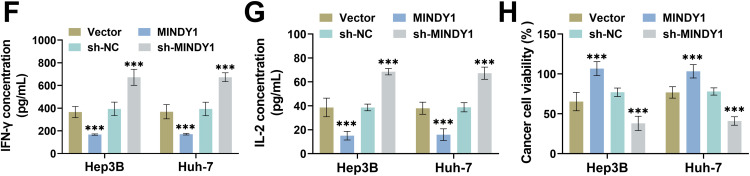
Overexpression of MINDY1 inhibits CD8^+^ T cell activation. (**A**) CFSE staining detected that the proliferation ability of CD8^+^ T cells was reduced after MINDY1 transfection, and was enhanced after sh-MINDY1 transfection. (**B**) Flow cytometry revealed CD8^+^ T cell apoptosis rate was elevated after MINDY1 transfection, and decreased after sh-MINDY1 transfection. (**C**) The cytotoxicity of CD8^+^ T cells was assessed through the LDH kit. (**D**,**E**) Flow cytometry confirmed that overexpression of MINDY1 caused a decrease in Perforin^+^ and TNF-α^+^ T cells, while knockdown of MINDY1 led caused a rise in these cells. (**F**,**G**) ELISA detected cytokines IFN-γ and IL-2 levels. (**H**) CCK-8 assay examined the survival rates of HCC cells. n = 3. *p < 0.05, **p < 0.01, ***p < 0.001

To investigate whether MINDY1 level in HCC cells affects CD8^+^ T cell functionality, CD8^+^ T cells were incubated with Hep3B and Huh-7 cells for 48 h. By CFSE staining, following co-culture with HCC cells that had been transfected with MINDY1, we observed a marked decline in CFSE-positive CD8^+^ T cells. In contrast, co-culture with HCC cells knocked down with MINDY1 caused a notable rise in CFSE-positive cells, demonstrating that overexpression of MINDY1 reduces the proliferative ability of CD8^+^ T cells (Fig. 4A). Overexpressing MINDY1 caused a markedly higher rate of apoptosis in CD8^+^ T cells, whereas knocking down MINDY1 caused the opposite result (Fig. 4B). After overexpressing MINDY1, the cytotoxicity of CD8^+^ T cells was notably reduced, while the opposite was true for knockdown of MINDY1 (Fig. 4C). Flow cytometry results demonstrated that overexpression of MINDY1 marked a decline in Perforin^+^ and TNF-α^+^ cell numbers, whereas knockdown of MINDY1 led to a notable rise in these cells, implying that MINDY1 overexpression inhibited CD8^+^ T cell activation (Fig. 4D,E). ELISA results demonstrated that overexpression of MINDY1 resulted in a marked decline in CD8^+^ T cytokines IFN-γ and IL-2 levels, whereas knockdown of MINDY1 caused a notable rise in these cytokines (Fig. 4F,G). Following co-culture with CD8^+^ T cells, MINDY1-transfected HCC cells showed markedly elevated viability, whereas MINDY1-knockdown cells exhibited reduced viability, suggesting that MINDY1 overexpression reduces the killing effect of CD8^+^ T cells (Fig. 4H). The data revealed that overexpression of MINDY1 resulted in diminished activity of CD8^+^ T cells, promoting immune escape in HCC cells.

### 3.5 MINDY1 Promotes PD-L1 Deubiquitination Through Wnt/***β***-Catenin Pathway

**Figure 5:**
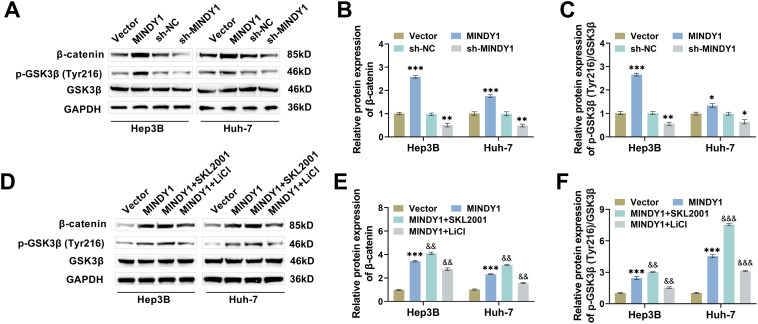

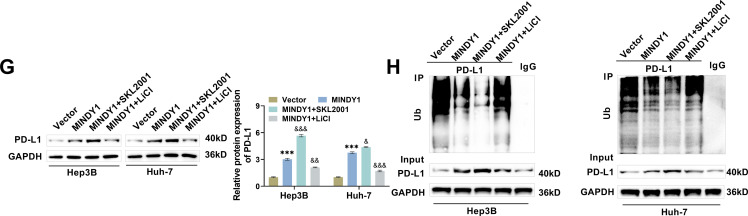
MINDY1 promotes PD-L1 deubiquitination through Wnt/β-catenin pathway. (**A–C**) MINDY1/Vector and sh-MINDY1/sh-NC were transfected, and Western blot analysis assessed that overexpression of MINDY1 elevated β-catenin and p-GSK3β(Tyr216)/GSK3β levels in HCC cells, and knockdown of MINDY1 did the opposite. (**D**–**F**) Western blot measured that SKL2001 (Wnt pathway activator) elevated the activating effect of overexpression of MINDY1 on the Wnt/β-catenin pathway, while pathway inhibitor LiCl decreased this phenomenon. **(G)** Western blot measured that SKL2001 increased PD-L1 protein expression, whereas LiCl led to PD-L1 downregulation. **(H)** Western blot examined that SKL2001 promoted the deubiquitination of PD-L1 in HCC cells, while LiCl suppressed its deubiquitination. n = 3. *p < 0.05, **p < 0.01, ***p < 0.001 vs. Vector/sh-NC; &*p* < 0.05, &&*p* < 0.01, &&&*p* < 0.001 vs. MINDY1

Research indicated that Wnt/β-catenin plays a vital role in PD-L1-mediated immunosuppression of tumor cells [25,38]. We hypothesized that MINDY1 may promote PD-L1 deubiquitination by modulating the Wnt/β-catenin pathway. To verify this speculation, we detected protein levels associated with this pathway by Western blot. After MINDY1 overexpression, β-catenin and p-GSK3β (Tyr216)/GSK3β levels were markedly upregulated, while the opposite was true when MINDY1 was knocked down, confirming that MINDY1 overexpression activated the Wnt/β-catenin pathway (Fig. 5A–C). Based on the overexpression of MINDY1, SKL2001 (a Wnt signaling pathway activator) treatment further increased β-catenin and p-GSK3β (Tyr216)/GSK3β levels, while the Wnt inhibitor LiCl reduced the levels of these proteins (Fig. 5D–F). SKL2001 treatment also further elevated PD-L1 expression, but LiCl caused the down-regulation of PD-L1 (Fig. 5G). SKL2001 further reduced PD-L1 ubiquitination level and promoted PD-L1 deubiquitination, while LiCl had the opposite effect (Fig. 5H). The findings mentioned earlier confirmed that MINDY1 promoted PD-L1 deubiquitination by triggering the Wnt/β-catenin pathway.

### 3.6 MINDY1 Hinders CD8^***+***^ T Cell Activation through Wnt/***β***-Catenin Pathway

**Figure 6:**
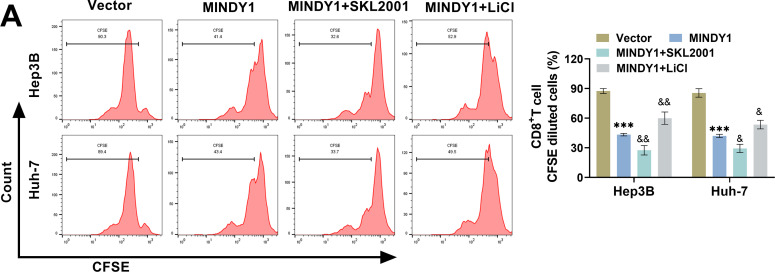

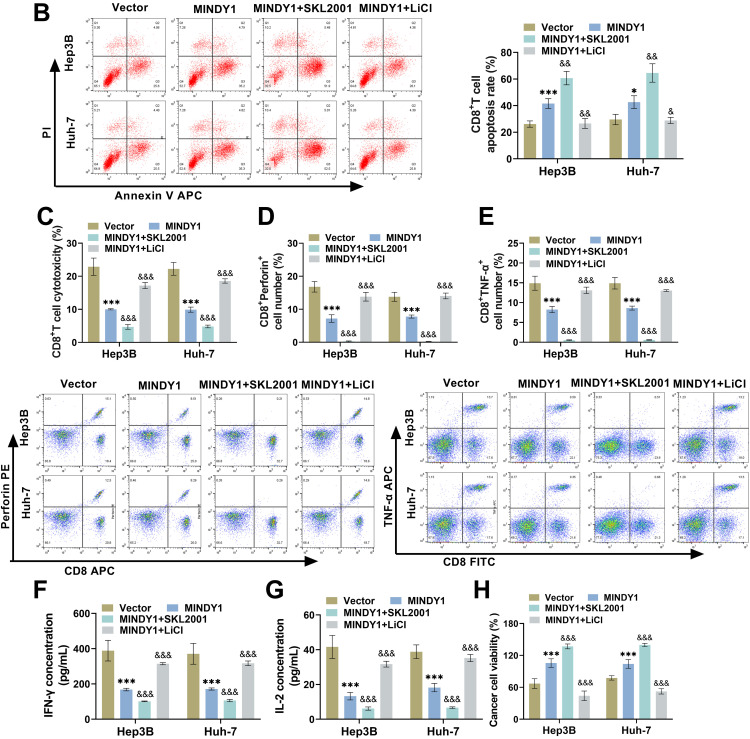
MINDY1 hinders CD8^+^ T cell activation through the Wnt/β-catenin pathway. (**A**) CFSE staining confirmed that co-culture with HCC cells transfected with MINDY1 resulted in diminished proliferation of CD8^+^ T cells, which was further diminished by SKL2001 treatment, whereas LiCl treatment did the opposite. (**B**) Flow cytometry indicated a rise in CD8^+^ T cell apoptosis after they were co-cultured with HCC cells overexpressing MINDY1, with an additional rise observed by SKL2001 treatment, while LiCl treatment reduced the apoptosis rate. (**C**) The cytotoxicity of CD8^+^ T cells was assessed through the LDH kit. (**D**,**E**) Flow cytometry confirmed that co-culturing with HCC cells overexpressing MINDY1 led to a decrease in Perforin^+^ and TNF-α^+^ T cells. After treatment with SKL2001, this decrease was further exacerbated, while LiCl increased activated CD8^+^ T cells. (**F**,**G**) ELISA was used to detect IFN-γ and IL-2 levels. (**H**) CCK-8 assay was employed to examine the survival rates of HCC cells. n = 3. *p < 0.05, ***p < 0.001 vs. Vector; &*p* < 0.05, &&*p* < 0.01, &&&*p* < 0.001 vs. MINDY1

Co-culture with HCC cells overexpressing MINDY1 caused a marked decline in the CFSE-positive and cytotoxic CD8^+^ T cells and a notable rise in apoptosis. Treatment with the Wnt signaling pathway activator SKL2001 further exacerbated CD8^+^ T-cell damage caused by overexpression of MINDY1, whereas the Wnt inhibitor LiCl attenuated CD8^+^ T-cell damage (Fig. 6A–C). TNF-α^+^ and Perforin^+^ T cells were markedly lower in the MINDY1+SKL2001 group, whereas the proportions were markedly higher in the MINDY1+LiCl group, suggesting that activating the Wnt/β-catenin pathway further inhibited the activation of CD8^+^ T cells (Fig. 6D,E). SKL2001 treatment resulted in a notable decline in IFN-γ and IL-2 levels, whereas LiCl caused a marked increase in these cytokines (Fig. 6F,G). Notably, after co-culture with CD8^+^ T cells, SKL2001 treatment further increased HCC cell viability, whereas LiCl treatment caused a marked decrease in cell survival (Fig. 6H). Wnt activator SKL2001 could further inhibit CD8^+^ T cell activation, in contrast to the Wnt inhibitor LiCl, which confirms that MINDY1 causes immune escape by triggering the Wnt/β-catenin pathway.

### 3.7 MINDY1 Induces HCC Tumor Growth and Immune Escape

**Figure 7:**
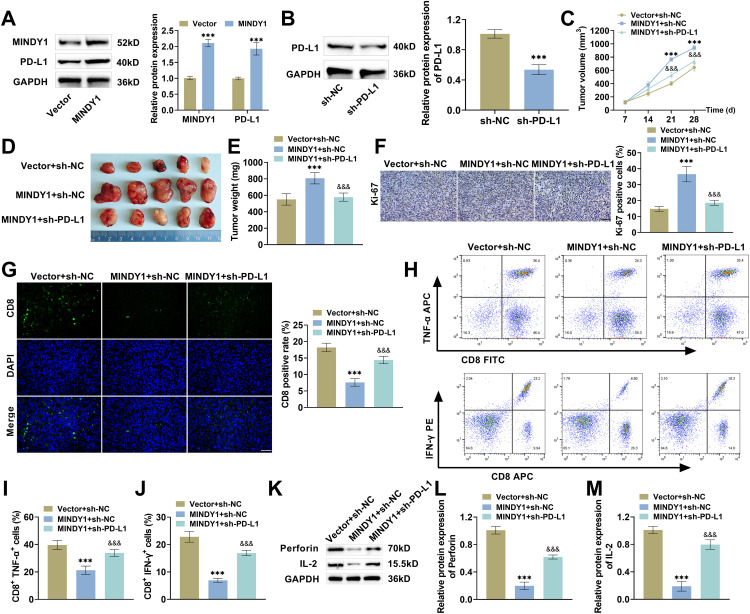
MINDY1 promotes tumor growth and immune escape. (**A**) Western blot revealed elevated MINDY1 and PD-L1 levels after MINDY1 overexpression. (**B**) Western blot examined a decline in PD-L1 levels after transfection with sh-PD-L1. (**C**) The sizes of the tumors were examined every week. On the 28th day, the tumors were removed and captured in photographs (**D**), and the tumor weights were also noted down (**E**). (**F**) Immunohistochemistry revealed that the Ki-67 level was elevated after transfection with MINDY1+sh-NC and declined after transfection with MINDY1+sh-PD-L1 (40×, 50 μM). (**G**) Immunofluorescence assay revealed that CD8-positive cells declined after transfection with MINDY1+sh-NC, and increased after transfection with MINDY1+sh-PD-L1 (40×, 50 μM). (**H**–**J**) Flow cytometry confirmed that CD8^+^TNF-α^+^ T cells and CD8^+^IFN-γ^+^ T cells percentages were decreased after transfection with MINDY1+sh-NC, and increased after transfection with MINDY1+sh-PD-L1. (**K**–**M**) Western blot indicated that transfection with MINDY1+sh-NC reduced the protein expressions of Perforin and IL-2 in tumor tissues, whereas transfection with MINDY1+sh-PD-L1 led to the upregulation of these two proteins. n = 5. ***p < 0.001 vs. Vector/sh-NC/Vector+sh-NC; &&&*p* < 0.001 vs. MINDY1+sh-NC

Ultimately, we developed a nude mouse subcutaneous xenograft tumor model to explore the impacts of MINDY1 on tumor immune escape. Injecting Hep3B cells transfected with MINDY1 notably increased MINDY1 and PD-L1 levels in the tumors of nude mice (Fig. 7A). After injecting Hep3B cells transfected with sh-PD-L1, the PD-L1 level was notably declined (Fig. 7B). This indicated that injecting Hep3B cells transfected with MINDY1 or sh-PD-L1 can effectively regulate MINDY1 or PD-L1 levels in tumors. The volume and mass of tumors in the MINDY1+sh-NC group of nude mice were markedly increased, indicating that overexpression of MINDY1 can promote tumor growth. While the volume and mass of tumors in the MINDY1+sh-PD-L1 group declined notably (Fig. 7C–E). Immunohistochemistry and immunofluorescence findings revealed that after overexpression of MINDY1, the positive rate of Ki-67 was markedly increased, while the positive rate of CD8 was decreased significantly. Silencing PD-L1 weakened the impact of MINDY1 overexpression (Fig. 7F,G). Additionally, overexpression of MINDY1 caused a marked decrease in TNF-α^+^ T cells and IFN-γ^+^ T cells, whereas silencing PD-L1 elevated the cells (Fig. 7H–J). After overexpression of MINDY1, Perforin and IL-2 protein levels in tumor tissues were decreased significantly, and silencing PD-L1 caused a rise in Perforin and IL-2 (Fig. 7K–M). The above results further indicated that overexpression of MINDY1 can induce HCC tumor growth and immune escape.

## 4 Discussion

HCC pathogenesis is extremely complex, involving the interaction of multiple factors, genes, and signaling pathways, and HCC is highly invasive and develops rapidly, leading to its early diagnosis and low survival rate [9,39]. Therefore, the search for the treatment targets of HCC could help prolong patient survival and increase the cure rate. As a vital part of the deubiquitinating enzyme family, MINDY1 possesses a unique structural domain that specifically recognizes and cleaves ubiquitin chains of specific linkages [40]. In a previous study, we demonstrated that MINDY1 modulates PD-L1 ubiquitination levels and promotes HCC malignant progression [19]. In this study, we examined MINDY1 levels in tumor tissues and HCC cells; MINDY1 was elevated in HCC, in agreement with findings from the TCGA database. In cell function studies, overexpression of MINDY1 promoted the malignant phenotypes of HCC cells, but knockdown of MINDY1 markedly suppressed these malignant biological behaviors. Findings from *in vivo* studies further confirmed that overexpression of MINDY1 promotes HCC growth, indicating that MINDY1 could act as a key target for HCC therapy.

Immune escape refers to the process by which tumor cells or pathogens are able to survive, proliferate, and spread in the body by evading the body’s immune system’s detection and attack [41,42]. Immune escape is vital in tumor development and has two main mechanisms of action [43,44]. The first is that tumor cells secrete immunosuppressive factors and induce the aggregation of immunosuppressive cells around the tumor, and decline the activity of immune cells [45]. The second is activating immune checkpoints PD-L1 and Programmed Death-1 (PD-1), induced PD-L1 binds to PD-1 on T cells’ surface, thereby suppressing T cell activation and proliferation [46]. Therefore, inhibition of PD-L1 expression is a vital direction for tumor immunotherapy and intervention. Previous studies have shown that increasing PD-L1 level in tumor-associated macrophages can reduce the number of activated CD8 T cells, thereby causing immune escape in breast cancer [47]. In our research, PD-L1 was elevated in HCC, and overexpression of MINDY1 increased PD-L1 levels, whereas silencing MINDY1 decreased PD-L1 levels. Notably, MINDY1 interacted with PD-L1 and promoted PD-L1 deubiquitination, enhancing PD-L1 stability. The study by Ren et al. also indicated that MINDY1 maintains PD-L1 stability, which in turn promotes immune escape of breast cancer [18]. These results suggested that MINDY1 may promote HCC advancement and immune escape via enhancing PD-L1 deubiquitination.

CD8^+^ T cells are a crucial class of lymphocytes in the immune system that express the CD8 molecule on their cell surface [48,49]. CD8^+^ T cells can release Perforin and granzyme, and also secrete tumor killing factors like TNF-α and IFN-γ, which specifically recognize and kill cancer cells, and can activate other immune cells [50]. To investigate the specific action of MINDY1 in the malignant advancement and immune escape of HCC, we co-cultured CD8^+^ T cells with HCC cells. MINDY1 overexpression inhibited CD8^+^ T cell proliferation, reduced the percentage of activated cells, declined IFN-γ and IL-2 levels, and decreased the killing impact of CD8^+^ T cells. This indicated that MINDY1 overexpression suppressed CD8^+^ T cells activation, thus weakening anti-tumor immunity mediated by CD8^+^ T cells. Taken together, we can reasonably conclude that overexpression of MINDY1 upregulates PD-L1 level and promotes its binding to PD-1, thereby suppressing CD8^+^ T cells activation, and thus inducing the immune escape of HCC.

Various investigations have indicated that the Wnt/β-catenin pathway affects PD-L1 levels and promotes PD-L1 deubiquitination, which in turn mediates tumor immune escape [25,51]. Wnt/β-catenin pathway is co-regulated by a variety of proteins, among which Wnt proteins are promoters and GSK-3β is a negative regulator [52]. When the function of Wnt protein is inhibited, the Wnt/β-catenin pathway is inactivated, and β-catenin is phosphorylated and then recognized, failing normal expression of target genes that rely on β-catenin activation, which then impacts the biological behavior of tumor cells [53]. Notably, in addition to regulating PD-L1 expression, the Wnt/β-catenin pathway is also responsible for regulating CD8^+^ T cell activation [54]. In our study, MINDY1 overexpression activated the Wnt/β-catenin pathway, whereas MINDY1 silencing had the opposite effect. Consequently, we hypothesized that MINDY1 enhances PD-L1 deubiquitination by stimulating this pathway, thus hindering CD8^+^ T cells activation. To verify this, we treated HCC cells overexpressing MINDY1 with inhibitors and activators of this pathway. The activator could further promote PD-L1 deubiquitination and inhibit CD8^+^ T cells activation, whereas the inhibitor had the opposite effect. Combining the above-mentioned studies, it was confirmed that MINDY1 promoted PD-L1 deubiquitination by stimulating the Wnt/β-catenin pathway, inhibited CD8^+^ T cells activation, and further promoted HCC immune escape.

Nevertheless, this study does have certain limitations. Nude mice have significantly lower numbers of CD8^+^ T cells *in vivo* and may not be the best model for assessing T cell-mediated immune responses. However, the presence of intact natural immunity (e.g., macrophages, NK cells) and adaptive immunity (T cells) in animals with normal immune systems rapidly recognizes and removes xenografted tumor cells (e.g., human tumor cells), leading to graft failure [55]. Therefore, animals with normal immune systems are also not suitable for direct xenotransplantation. However, in the future, a homologous transplantation model can be used to further validate in depth the role of MINDY1 in regulating immune escape from HCC, while ensuring an intact immune system and no rejection. Furthermore, the CD8^+^ T cells from healthy donors may not be able to fully replicate the complex conditions of the HCC tumor microenvironment. In future studies, we will use the T cells of HCC patients to further verify the role of MINDY1 in HCC immune escape. Additionally, this study solely focused on the role of CD8^+^ T cells in the immune escape mediated by MINDY1, and did not systematically explore the potential contributions of other key immune cells (like NK cells, CD4^+^ T cells, and B cells). Therefore, in the future, it will be possible to explore the roles of other immune cells in the MINDY1-mediated immune escape of HCC. In the future, it will also be possible to co-culture MINDY1-overexpressing tumor cells with CD8^+^ T cells and treat them with PD-L1 blocking antibodies to verify whether changes in the ubiquitinylation status of PD-L1 directly affect the function of CD8^+^ T cells. The cellular signaling pathways are highly intricate. In the future, other pathways activated by MINDY1 should be explored to further clarify its mechanism of action.

## 5 Conclusion

Overexpression of MINDY1 promotes PD-L1 deubiquitination, hinders CD8^+^ T cells activation, and further promotes the malignant progression of HCC tumors. Further mechanistic studies showed that MINDY1 acts by stimulating the Wnt/β-catenin pathway. This research clarifies the mechanism by which MINDY1 facilitates the immune escape of HCC, and confirms that MINDY1 is a promising target for HCC therapy.

## Data Availability

The data supporting the findings of this study can be obtained from the corresponding author upon request.

## Abbreviations

MINDY1: Motif interacting with ubiquitin-containing novel DUB family-1
PD-L1: Programmed death-ligand 1
HCC: Hepatocellular carcinoma
CCK-8: Cell Counting Kit-8
TCGA: The Cancer Genome Atla
PBMCs: Peripheral blood mononuclear cells
TNF-α: Tumor necrosis factor-α
CFSE: Carboxyfluorescein succinimidyl ester
BSA: Bovine Serum Albumin
LDH: Lactate dehydrogenase
IFN-γ: Interferon-gamma
IL-2: Interleukin-2
IgG: Immunoglobulin G
Co-IP: Co-immunoprecipitation
